# Trajectories of brain development in school-age children born preterm with very low birth weight

**DOI:** 10.1038/s41598-018-33530-8

**Published:** 2018-10-22

**Authors:** K. Sripada, K. J. Bjuland, A. E. Sølsnes, A. K. Håberg, K. H. Grunewaldt, G. C. Løhaugen, L. M. Rimol, J. Skranes

**Affiliations:** 1 Department of Clinical & Molecular Medicine, Norwegian University of Science & Technology, Trondheim, Norway; 2 Department of Pediatrics, Sørlandet Hospital, Arendal, Norway; 3 Department of Neuromedicine & Movement Science, Norwegian University of Science & Technology, Trondheim, Norway; 4 Department of Radiology & Nuclear Medicine, St. Olav’s Hospital, Trondheim, Norway; 5 Department of Pediatrics, St. Olav’s Hospital, Trondheim, Norway; 6 Department of Circulation & Medical Imaging, Norwegian University of Science & Technology, Trondheim, Norway

## Abstract

Preterm birth (gestational age < 37 weeks) with very low birth weight (VLBW, birth weight ≤ 1500 g) is associated with lifelong cognitive deficits, including in executive function, and persistent alterations in cortical and subcortical structures. However, it remains unclear whether “catch-up” growth is possible in the preterm/VLBW brain. Longitudinal structural MRI was conducted with children born preterm with VLBW (n = 41) and term-born peers participating in the Norwegian Mother and Child Cohort Study (MoBa) (n = 128) at two timepoints in early school age (mean ages 8.0 and 9.3 years). Images were analyzed with the FreeSurfer 5.3.0 longitudinal stream to assess differences in development of cortical thickness, surface area, and brain structure volumes, as well as associations with executive function development (NEPSY Statue and WMS-III Spatial Span scores) and perinatal health markers. No longitudinal group × time effects in cortical thickness, surface area, or subcortical volumes were seen, indicating similar brain growth trajectories in the groups over an approximately 16-month period in middle childhood. Higher IQ scores within the VLBW group were associated with greater surface area in left parieto-occipital and inferior temporal regions. Among VLBW preterm-born children, cortical surface area was smaller across the cortical mantle, and cortical thickness was thicker occipitally and frontally and thinner in lateral parietal and posterior temporal areas. Smaller volumes of corpus callosum, right globus pallidus, and right thalamus persisted in the VLBW group from timepoint 1 to 2. VLBW children had on average IQ 1 SD below term-born MoBa peers and significantly worse scores on WMS-III Spatial Span. Executive function scores did not show differential associations with morphometry between groups cross-sectionally or longitudinally. This study investigated divergent or “catch-up” growth in terms of cortical thickness, surface area, and volumes of subcortical gray matter structures and corpus callosum in children born preterm/VLBW and did not find group × time interactions. Greater surface area at mean age 9.3 in left parieto-occipital and inferior temporal cortex was associated with higher IQ in the VLBW group. These results suggest that preterm VLBW children may have altered cognitive networks, yet have structural growth trajectories that appear generally similar to their term-born peers in this early school age window.

## Introduction

Cognitive deficits among individuals born preterm (gestational age < 37 weeks) with very low birth weight (VLBW, birth weight ≤ 1500 g) can persist for decades^1–6^. Executive functions, which are foundational for academic performance and quality of life, are often impaired in the preterm-born VLBW population, even among those with otherwise typical cognitive ability^7–9^, starting in early childhood^10^ and lasting into adulthood^11–14^. Cognitive impairment following preterm birth may derive from altered connectivity that begins *in utero*^15^, as suggested by a recent fetal resting state functional magnetic resonance imaging (MRI) finding of reduced connectivity in a left hemisphere proto-language region^16^.

What is unclear is whether these structural and functional differences can potentially diminish over time (“catch up”), or whether they will persist. Finding a window for catch up development could be a therapeutic opportunity, yet there is limited evidence for catch-up growth in this population. A small number of studies have identified differential growth in corpus callosum volume^17^, cerebellar volume^18^, and cortical thickness in specific regions^19^. By contrast, much research has pointed to altered brain growth following preterm birth/VLBW^20–22^ and similar brain growth rates for preterm/VLBW and term-born children and adolescents, despite different starting points^23–26^. Smaller volumes, decreased general cognitive functioning, and altered frontal, thalamo-cortical, and subcortical connectivity are typical in this population^27–29^. Adolescents born extremely preterm/VLBW have shown a 1.6-year younger “brain age” based on *T*_1_-weighted whole brain structural data, compared to adolescents born after gestational week 29^30^.

As preterm-born children enter school age, they have been reported to have increased distractibility, worse inhibitory control, and poorer executive function skills that may contribute to poorer social competence^31,32^. A clearer understanding of the timing and extent of structural and functional plasticity in the preterm brain – and the potential for catch-up development – is thus needed^33,34^. Cross-sectional findings from an overlapping sample of this preterm/VLBW cohort and term-born participants in the Norwegian Mother and Child Cohort Study (MoBa)^35,36^ identified smaller cortical surface area bilaterally in frontal, temporal, and parietal lobes; thicker cortex in frontal and occipital regions; thinner cortex in posterior parietal areas; reduced volumes of subcortical structures including corpus callosum and hippocampus in the preterm/VLBW group; and only limited group differences in white matter tracts. This study is the first to present longitudinal findings comparing the preterm/VLBW and MoBa cohorts.

The aim of this study was therefore to determine whether the cortical and subcortical deviations found at the first timepoint^35,36^ persisted longitudinally, and whether VLBW children showed different growth trajectories of brain structures compared to term-born peers. To our knowledge, this study is the first to investigate longitudinal morphometric changes in the preterm brain at early school age. Moreover, we assessed executive function at early school age and examined possible interactions with brain development over time, in this sensitive window where demands on executive function escalate^37^. We expected that preterm-born/VLBW children would continue to show altered brain structure, as well as associations between MRI findings and both cognitive scores and perinatal morbidity markers.

## Methods

### Participants

Preterm-born VLBW participants (n = 41) born between 2003 and 2007 were recruited based on admittance to the Neonatal Intensive Care Unit at St. Olav’s University Hospital in Trondheim, Norway. Term-born control participants from central Norway (n = 128) born between 2001 and 2007 were recruited from the national Norwegian Mother and Child Cohort Study (MoBa) study, coordinated by the Norwegian Institute of Public Health^38,39^. Cerebral MRI and cognitive data were collected at two timepoints in childhood: first at mean age 8.0 years (range: 4.9–10.6) then at mean age 9.3 years (range: 6.1–12.0).

Exclusion criteria were severe cerebral palsy (unable to complete neuropsychological testing and MRI), severe sensory impairments, and/or MRI contraindications. Birth weight and gestational age for MoBa participants were retrieved from registry data (not available for 2 participants, for whom parent-reported birth weight was used); birth weight > 2500 g and gestational age ≥ 37 completed weeks were inclusion criteria for term-born participants in this study. Among the VLBW participants, five children with retinopathy of prematurity, one with epilepsy and mild cerebral palsy, one with intraventricular hemorrhage (grade 1) and mild cerebral palsy, and 2 others without cerebral palsy with intraventricular hemorrhage (grades 1 and 3) who successfully completed the neuropsychological assessments and MRI were included in the analyses; IQ range of these participants was 87 to 117. Four participants (2 VLBW and 2 term-born) had ADHD, and one term-born child had a history of concussion. Overall 120 participants (VLBW n = 30) had two successful MRI scans, and 49 (VLBW n = 11) with only one successful scan were also included in this study. Six preterm participants and no controls had twins. Morphometry findings at the first timepoint in an overlapping sample of this cohort were previously reported using the FreeSurfer cross-sectional processing stream^35,36^. Birth weight, gestational age, Apgar scores at 1 and 5 minutes, number of days in the NICU, and number of neonatal days on ventilator were the clinical variables in the VLBW group assessed for partial correlations with MRI data.

### MRI

MRI data were collected using a 12-channel head coil on a 1.5 T Siemens Avanto scanner (Siemens, Erlangen, Germany). The total scan time was on average 30 minutes. The pulse sequence used for morphometric analyses was a 3D *T*_1_-weighted magnetization prepared rapid acquisition gradient echo (MPRAGE) scan with the following parameters: TR = 2400 ms, TE = 3.61 ms, TI = 1000 ms; flip angle 8°, FOV 240 × 240 mm^2^, and TA = 4 minutes and 18 minutes. Each volume consisted of 160 sagittal slices with voxel sizes of 1.25 × 1.25 × 1.20 mm^3^. All subjects had between one and four MPRAGE *T*_1_ scans. Each MPRAGE series was visually inspected using FreeSurfer’s tkregister2 tool and Aeskulap Viewer (http://aeskulap.nongnu.org) to identify artifacts and evaluate Talairach alignment, and only scans with no or minimal movement artifacts were included. The FreeSurfer package QA Tools was run on all subjects for visual inspection of segmentation. Ten subjects did not have any satisfactory MPRAGE *T*_*1*_–weighted scans due mostly to motion artifacts or other objects such as braces.

### Image analysis

All image analysis was performed with the freely available FreeSurfer image analysis suite version 5.3.0 (http://surfer.nmr.mgh.harvard.edu). The technical details of the FreeSurfer image processing procedures are described in prior publications^40–55^. Images in this study were processed automatically using FreeSurfer’s longitudinal stream to extract reliable volume and thickness estimates across the timepoints^55^. Specifically, an unbiased within-subject template image was created using robust, inverse consistent registration^54^. Several processing steps, such as skull stripping, Talairach transforms, atlas registration as well as spherical surface maps and parcellations were then initialized with common information from the within-subject template, significantly increasing reliability and statistical power^55^. The subcortical brain structures included in the analyses are based on the automated segmentation and labeling procedure in FreeSurfer^46,48^. The cortical parcellation scheme in FreeSurfer by Desikan *et al*.^50^ was used for the table in Section 3.4, and the naming based on the Destrieux *et al*.^53^ cortical parcellation scheme was used to provide additional detail in the text. Ventricular system volume is the aggregate of lateral, inferior, third, and fourth ventricle volumes.

We used a method described by Hansen and Brezova *et al*.^56^ to measure intracranial volume (ICV). Briefly, ICV was estimated with an automated reverse brain mask method using the “new segment” approach of the SPM8 toolbox (release 5236) (www.fil.ion.ucl.ac.uk/spm) inside the cranium, including the brain, meninges, and cerebrospinal fluid. The pituitary gland is excluded by a straight line through the upper pituitary stalk in the axial plane. The lowest point of the cerebellum defines the caudal border. All ICV segmentations were visually inspected, and none were rejected or manually adjusted.

### Cognitive measures

Comprehensive neuropsychological assessment and IQ testing were performed in the two groups. At neuropsychological assessment, parents reported whether children had received or planned to receive special education, such as aid of an assistant or help with specific subjects, at school/preschool. Executive function scores deemed invalid or incomplete by test administrators were not included in the analyses, leading to different participant numbers for the different tests.

#### IQ in VLBW group

In the VLBW group, children ≥ 6 years of age were assessed with Wechsler Intelligence Scale for Children, fourth edition (WISC-IV)^57^, which comprises four indices: Verbal Comprehension Index, Perceptual Reasoning Index, Working Memory Index, and Processing Speed Index. Children < 6 years of age were assessed with the complete version of the Wechsler Preschool and Primary Scale of Intelligence, third edition (WPPSI-III)^58^. Since most participants were tested twice, scores from their first cognitive assessment were used here to avoid practice effects; however, for 11 VLBW participants, IQ scores from the first timepoint were not available (e.g., due to incomplete testing), so scores from the second timepoint were used instead. Due to incomplete testing, Verbal Comprehension Index was used as a substitute for Full-scale IQ Index for four participants, and Perceptual Reasoning Index was used as a substitute for one participant. Full-scale IQ Index scores were used for this study.

#### IQ in control group

Cognitive abilities in the controls who were ≥ 6.5 years of age were assessed with the Wechsler Abbreviated Scale of Intelligence (WASI)^59^. WASI is a validated screening test used to assess verbal knowledge, visual information processing, spatial and nonverbal reasoning, and general intelligence. Three IQ scores can be measured using the WASI: Verbal IQ and Performance IQ, which when combined provide an estimated Full-scale IQ score. The controls < 6.5 years of age completed a short form of the WPPSI-III^58^, including the vocabulary, similarities, block design, and matrices subtests. Full-scale IQ Index scores from timepoint 1 were used for this study.

#### NEPSY Statue

The NEPSY Statue subtest of the Developmental NEuroPSYchological Assessment, Norwegian version (NEPSY)^60^, is designed to assess motor control and inhibition by asking the child to maintain a body position for 75 seconds and ignore distracting sounds that they are not informed about before the test starts. Points are awarded per five-second interval: two points for full response inhibition, one point for one inappropriate response, and zero points for more than one inappropriate response^61^. This study used raw scores, where a higher score reflects better response inhibition.

#### Spatial Span

The Spatial Span subtest of the Wechsler Memory Scale, third edition (WMS-III)^62^, is designed to evaluate visual working memory. The examiner points to blue blocks on a white board and asks the participant to point to the blocks in the same order, with increasing difficulty. Later, the participant is instructed to point in reverse order, also with increasing difficulty. For this study, we used the raw total outcome score of correctly replicated items.

### Socio economic status

Hollingshead’s^63^ two factor index of social position based on education and occupation of one parent or the mean index of both parents was used to calculate socioeconomic status.

### Statistical analysis

IBM SPSS 24 (Chicago, USA) was used to evaluate group differences and correlations between demographic, clinical, morphometric, and cognitive measures, with significance threshold at *p* < 0.05. One-way ANOVA was used to compare demographic variables with normal distribution, with *p* < 0.05 indicating significant group differences. Mann-Whitney U Test was used for age at scan, which had a nonparametric distribution as assessed by Shapiro-Wilk’s test (*p* < 0.05). Chi-square (χ^2^) testing for association was used for socioeconomic status. Drop-out analysis used independent samples *t*-tests within the preterm and term-born groups in terms of gestational age, birth weight, receiving help at school, age at scan, and IQ, based on the 14 term-born participants and 12 preterm/VLBW participants who met for assessment and were excluded from this analysis.

Longitudinal analyses of changes in cortical morphometry from timepoint 1 to 2 were run in Matlab 2015b (MATLAB and Statistics Toolbox Release 2015b. The MathWorks, Inc., Natick, Massachusetts, USA) by adapting the linear mixed effects module in FreeSurfer 5.3.0^64^. A linear mixed effects model was fitted in each location (vertex) across the reconstructed cortical surface, with cortical area or cortical thickness as the dependent variable; intercept, time from baseline, age at baseline, group, sex, and interaction (group × time) as independent variables; and intercept as random factor. Using these variables, contrast vectors were set in order to test for an interaction effect between group and time and for each of the executive function tests and IQ. Effects of time were assessed within each group for each of the cognitive scores.

General linear models were fitted in Matlab 2015b for cross-sectional cortical thickness and surface area analyses for both timepoints, controlled for age at scan and sex. General linear models were also fitted to assess relationships between cortical surface area or thickness with IQ and executive function scores, between and within groups, at each timepoint. General linear models for both cortical surface area and thickness were also fitted at timepoint 2 with cortical measure (area or thickness) as the dependent variable and group as the independent variable, co-varying for sex, age at scan, and retinopathy of prematurity. General linear models in SPSS were fitted for cross-sectional group comparisons of subcortical brain structure volumes, controlled for ICV, age at scan, and sex; analysis of ICV controlled only for age at scan and sex.

To correct for multiple comparisons, the *p*-maps from left and right hemisphere were combined and thresholded to yield an expected false discovery rate (FDR) of 5% across both hemispheres. In order to generate effect size maps that are comparable across the morphometry variables to investigate annualized rate of change, cortical area and cortical thickness were log-transformed prior to fitting the model to the data; the beta value for time, resulting from the model fit, was back-transformed and multiplied by 100 in order to obtain percent change per unit of time^26^. Brain figures display MRI data overlaid on the FreeSurfer fsaverage white surface.

Holm–Bonferroni step-down^65^ was used to correct for multiple comparisons for tests of group differences and correlations in subcortical volumes based on 24 structures compared and α = 0.05. Partial correlation tests, controlled for age at scan, sex, and ICV were used to investigate the relationships between subcortical volumes and cognitive and perinatal data. Raw cognitive test scores were adjusted for age.

### Ethics

The Regional Committee for Medical Research Ethics approved the study protocol (project number 2010/2359), and written, informed consent was obtained from the parents/guardians of all participants. The study was performed in accordance with relevant guidelines and regulations.

## Results

### Clinical profile

Demographic and clinical characteristics of the two groups are presented in Table 1. VLBW participants had lower IQ by approximately 1 SD, were more likely to be receiving or plan to receive help at school or preschool based on parent report (36.6% vs 7.3%), and were younger than term-born peers by approximately 6 months at both scans. There was no statistically significant association between socioeconomic status and group (χ^2^(1) = 7.39, *p* = 0.12). Average time between scans was 14.3 months for VLBW participants and 16.0 months for term-born peers. Drop-out analysis did not reveal any significant differences within either group in terms of gestational age, birth weight, receiving help at school, age at scan, or IQ.

**Table 1:** Demographic and clinical profile of the two study groups.

	VLBW (n = 41)			Term-born (n = 128)			*p*-value
	Mean	SD	Range	Mean	SD	Range	
Birth weight, grams	1039	313	416–1495	3679	529	2510–5460	<0.0001*
Gestational age, weeks (days)	29(1)	2(6)	23(4)–35(1)	40(0)	1(2)	37(1)–42(4)	<0.0001*
Age, years							
Timepoint 1	7.7	1.7	(5.0, 10.4)	8.2	1.2	(4.9, 11.1)	0.51
Timepoint 2 (n = 30 VLBW, 90 term-born)	8.9	1.7	(6.1, 10.7)	9.5	1.2	(6.3, 12.0)	0.31
Sex, male:female	17:24			66:62			0.26
IQ	93.5	9.8	(74, 117)	107.4	13.8	(73, 139)	<0.0001*
Socioeconomic status (low 1, high 5; n = 37 VLBW, 109 term-born)	3.9	0.9	(1, 5)	4.3	0.9	(1,5)	0.12
Received/plan to receive help at school, **(%)**	15 (36.6)			9 (7.0)			<0.0001*

Significant group differences indicated with *. *Abbreviations:* SD, standard deviation; VLBW, very low birth weight.

### Executive function

Scores on the two executive function tests are presented in Table 2 with significance testing and at each timepoint and for longitudinal group × time effects. WMS-III Spatial Span was significantly worse in the VLBW group at timepoint 2 (*d* = −0.62, *p* = 0.005).

**Table 2:** Scores on NEPSY Statue and WMS-III Spatial Span assessments, tested for group differences at each timepoint and for effect of group × time.

Assessment	Timepoint	n, VLBW, control	VLBW mean ± SD	Term-born mean ± SD	*d*	*p*-value	LME *p*-value
Statue	1	n = 26, 127	28.4 ± 3.1	27.6 ± 3.1	0.25	0.25	0.21
	2	n = 29, 90	28.5 ± 2	28.7 ± 1.9	−0.13	0.55	
Spatial span	1	n = 26, 128	11.7 ± 2.9	12.6 ± 2.9	−0.32	0.14	0.51
	2	n = 29, 87	12.1 ± 2.9	13.9 ± 2.9	−0.62	0.005*	

Group differences tested using the general linear model, controlled for age, shown with effect size and number of participants included in each analysis. Statistically significant results are denoted by *. LME *p*-value refers to the longitudinal interaction analyses (score × time). *Abbreviations:* CI: confidence interval; *d:* Cohen’s *d*; LME: linear mixed effects; VLBW: very low birth weight.

### Subcortical volumes

#### Group differences: longitudinal and cross-sectional

**Figure 1:**
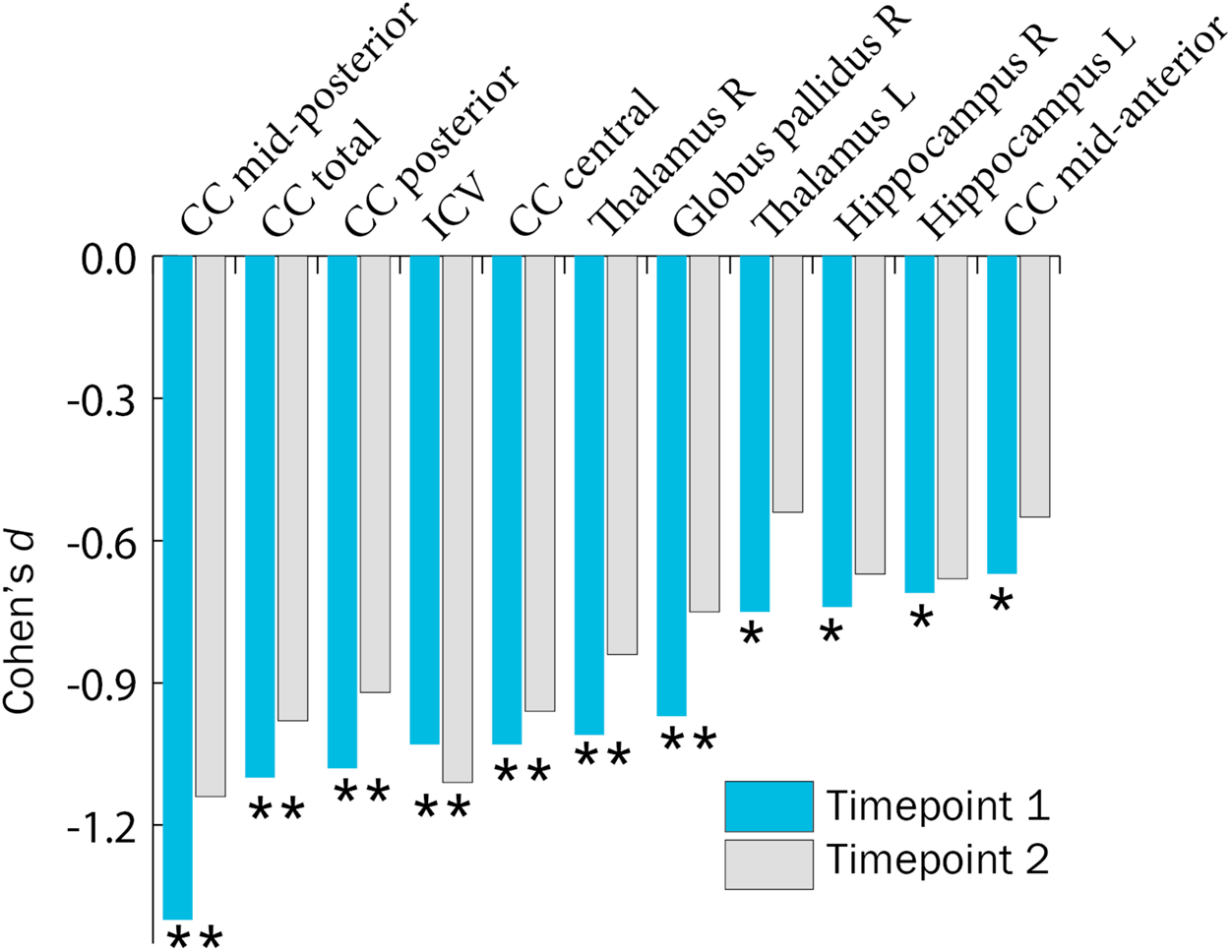
Subcortical structures showing significant group differences (indicated by *) at timepoint 1 and timepoint 2, in right (R) and left (L) hemispheres, shown with effect size of VLBW group compared to controls. Volumes adjusted for sex, age at scan, and ICV (ICV itself only adjusted for sex and age at scan). Abbreviations: CC: corpus callosum; ICV: intracranial volume; VLBW: very low birth weight.

Group differences in subcortical gray matter structures, corpus callosum volume, and ICV are presented in Supplemental Table 1, and effect sizes for structures showing significant group differences in Figure 1. Longitudinal analyses of subcortical brain structure volumes did not identify any group × time effects. Post-hoc removal of ICV as a covariate also did not identify any longitudinal group differences. Corpus callosum (central, mid-posterior, and posterior segmentations, and total corpus callosum volume), right globus pallidus, and right thalamus were significantly smaller in the VLBW group compared to controls at both timepoints. The ventricular system was significantly larger in the VLBW group at both timepoints. In addition, group differences in bilateral hippocampus, left thalamus, and corpus callosum mid-anterior subsegmentation size were significant at timepoint 1, with the VLBW group showing smaller volumes.

#### Volume-cognition relationships

No group × score interactions (for executive function scores, IQ, or receiving help at school) were found for any of the subcortical volumes after correction for multiple comparisons. Several associations were found within the groups. In the VLBW group at timepoint 2, larger corpus callosum volume (total volume and posterior, mid-posterior, and central subsegmentations) was negatively associated with receiving help/special education at school, although only the posterior segment structure-function relationship was significant after correction for multiple comparisons (*p* = 0.00089). Among controls, ventricular system volume was significantly correlated with receiving/planning to receive help at school at timepoint 1 (R = 0.45, *p* < 0.001) and timepoint 2 (R = 0.31, *p* = 0.0011), and left nucleus accumbens volume was correlated with NEPSY statue score at timepoint 1 (R = 0.33, p = 0.001).

#### Volume-perinatal health data relationships in VLBW group

Birth weight was positively correlated with left thalamus volume at timepoint 1 (R = 0.67, *p* = 0.002). Right hippocampus volume at timepoint 2 was negatively associated with Apgar 5 minute score (R = −0.71, *p* = 0.00091).

### Group differences in cortical structure

#### Longitudinal changes

No evidence was found for longitudinal group × time interactions in either cortical thickness or surface area development between the two scanning timepoints (approximately 16 months apart).

#### Cross-sectional findings

Cross-sectional group differences in cortical thickness and surface area were widespread across the cortical mantle at timepoint 2 (Table 3). Surface area group differences were more global than those for cortical thickness. Cortical thickness was increased in the VLBW group frontally and decreased in parietal and temporal regions. Similar morphometry findings at timepoint 1 were previously reported using cross-sectional processing in an overlapping sample in this cohort^35^.

**Table 3:** Proportion (%) of each cortical parcellation showing significant differences in cortical surface area and thickness between VLBW and controls for both timepoints.

Cortical parcellation	Cortical thickness				Surface area			
	Timpoint 1		Timepoint 2		Timpoint 1		Timepoint 2	
	Left	Right	Left	Right	Left	Right	Left	Right
Banks of the superior temporal gyrus	24	13	57	49	100	100	100	100
Caudal anterior cingulate gyrus	93	0	0	0	100	100	100	100
Caudal middle frontal gyrus	0	3	0	0	100	100	100	100
Cuneus	65	36	65	20	100	93	100	71
Entorhinal cortex	0	0	0	0	26	0	23	2
Frontal pole	100	0	100	0	100	100	100	100
Fusiform gyrus	10	13	4	12	85	85	84	80
Inferior parietal gyrus	7	15	55	14	99	100	84	99
Inferior temporal gyrus	3	29	15	34	41	100	57	100
Insula	1	25	1	2	98	25	98	25
Isthmus cingulate	10	39	2	35	100	100	100	100
Lateral occipital gyrus	50	58	43	59	74	78	60	82
Lateral orbitofrontal gyrus	54	33	22	13	100	96	100	96
Lingual gyrus	35	43	39	50	100	96	100	97
Medial orbitofrontal gyrus	99	2	66	6	100	100	100	100
Middle temporal gyrus	40	25	64	34	85	85	90	85
Paracentral gyrus	0	0	0	0	52	100	7	7
Parahippocampal gyrus	0	6	0	23	100	50	100	42
Pars opercularis	14	46	0	24	100	97	100	82
Pars orbitalis	0	78	0	56	100	100	100	100
Pars triangularis	0	100	0	95	100	100	100	70
Pericalcarine sulcus	59	37	100	32	100	46	100	29
Postcentral gyrus	0	0	0	0	100	61	98	27
Posterior cingulate	43	0	6	0	100	100	100	100
Precentral gyrus	0	0	0	0	97	95	79	85
Precuneus	0	4	7	11	100	95	82	85
Rostral anterior cingulate	100	0	27	0	100	100	100	100
Rostral middle frontal gyrus	37	31	3	0	85	82	76	59
Superior frontal gyrus	28	3	21	0	84	100	74	94
Superior parietal gyrus	11	12	5	17	93	100	73	78
Superior temporal gyrus	1	4	3	4	100	18	100	18
Supramarginal gyrus	12	15	44	7	99	64	82	56
Temporal pole	0	0	0	0	11	9	32	7
Transverse temporal gyrus (Heschl’s gyrus)	0	0	0	0	100	0	100	0

This table presents the percentage of surface area in atlas space (fsaverage) that showed a significant group difference between the VLBW and control groups in each of the 36 cortical parcellations in the Desikan-Killiany parcellation scheme^50^, for both surface area and thickness for each hemisphere at each timepoint after 5% FDR correction. *Abbreviations*: FDR: false discovery rate; VLBW: very low birth weight.

#### Surface area

**Figure 2:**
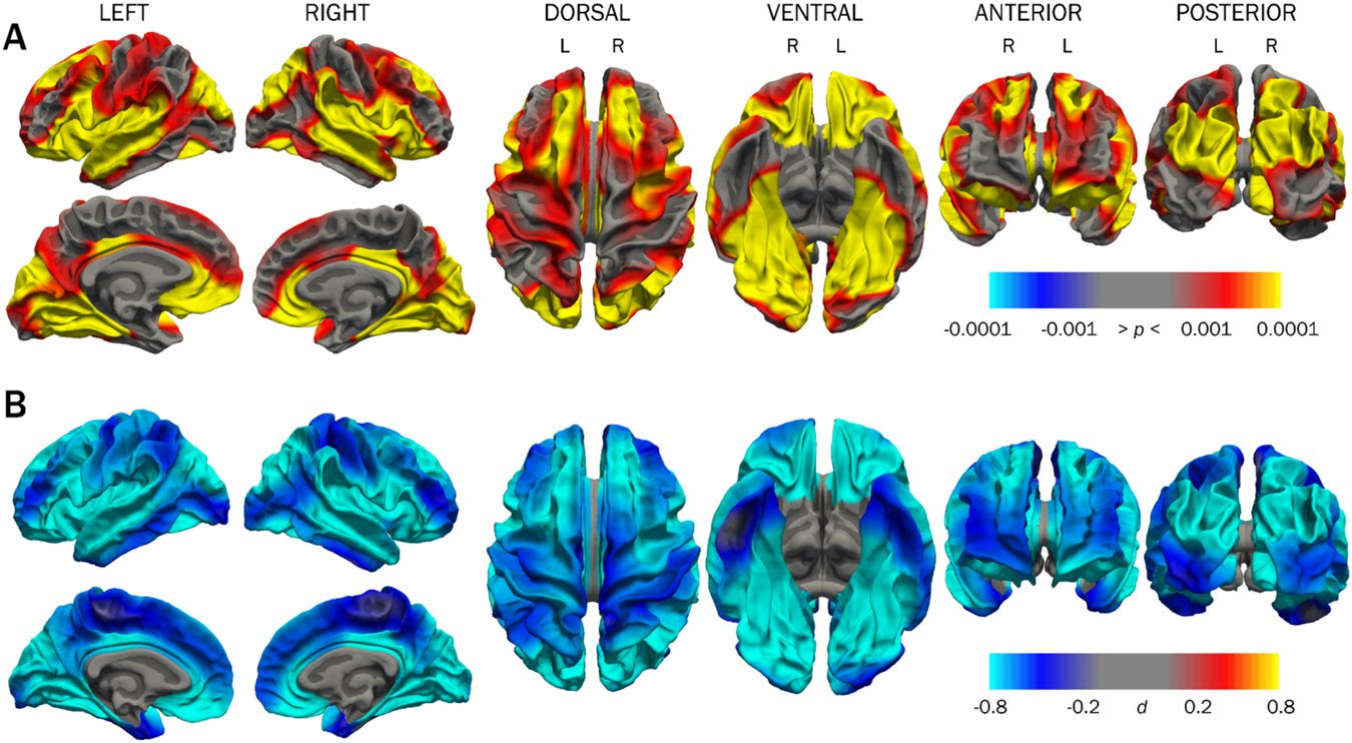
Cortical surface area group differences between VLBW and control groups at timepoint 2. Row A shows *p-*maps and row B shows effect size. The *p-*maps were produced from GLM models fitted at each location (vertex) across the cortical surface, with cortical area as the dependent variable and group as the independent variable, co-varying for sex and age at scan. The *p*-maps were thresholded to yield an expected 5% FDR across both hemispheres. In the effect size maps, blue represents areas of reduced surface area in the VLBW. *Abbreviations*: *d*: Cohen’s *d*; FDR: false discovery rate; VLBW, very low birth weight.

Group differences in surface area were widespread, shown in Figure 2 and Table 3 for timepoint 2, and were more widespread than group differences in cortical thickness. There were no cortical areas with larger surface area in the VLBW group, as indicated by Cohen’s *d* values (Figure 2B, range: −1.37 > d < −0.054 on left; −1.50 > d < −0.0075 on right). Cortical surface area results from timepoint 1 in this study using the longitudinal processing stream are presented in Supplementary Figure 1.

#### Cortical thickness

**Figure 3:**
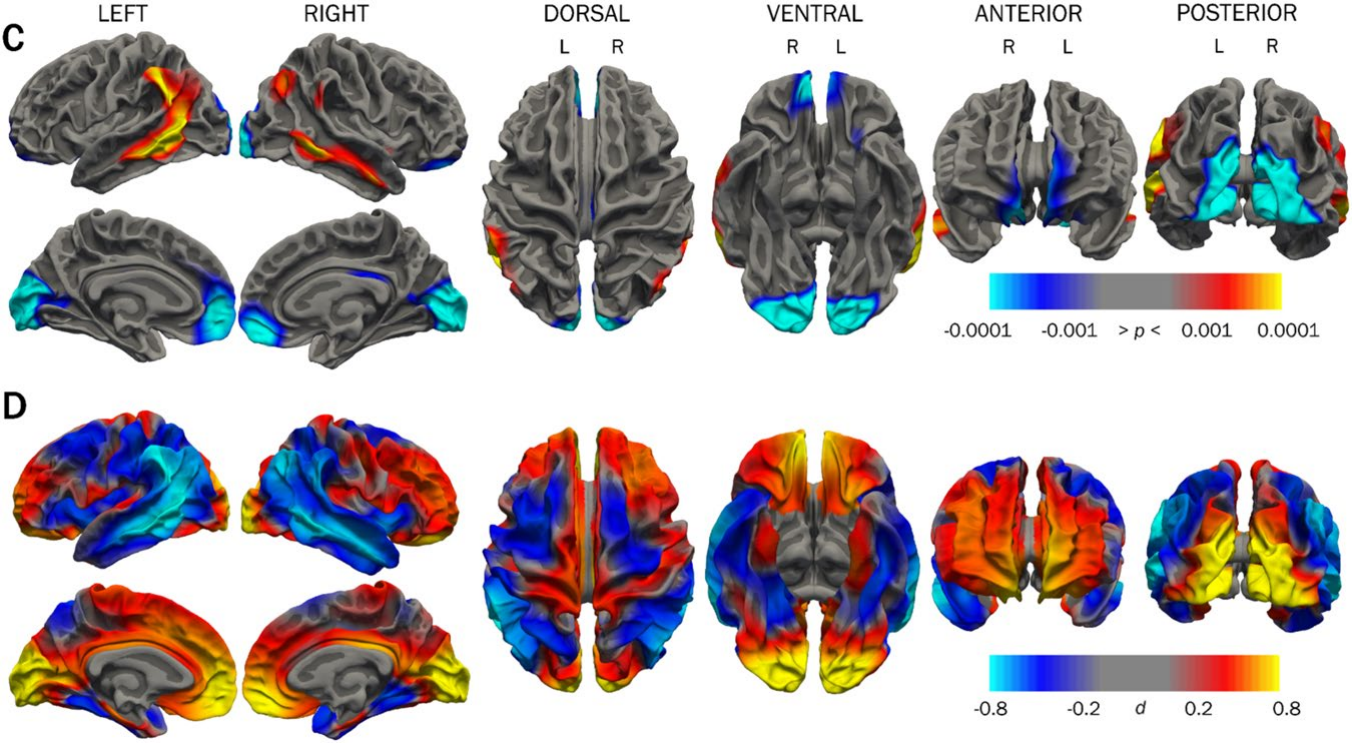
Cortical thickness group differences between VLBW and control groups at timepoint 2. Row C shows *p-*maps and row D shows effect size. The *p-*maps were produced from GLM models fitted at each vertex across the cortical surface, with cortical thickness as the dependent variable and group as the independent variable, co-varying for sex and age at scan. The *p*-maps were thresholded to yield an expected 5% FDR across both hemispheres. In the effect size maps, red-yellow color represents areas of increased thickness in the VLBW, while blue represents cortical thinning. *Abbreviations*: *d*: Cohen’s *d*; FDR: false discovery rate; VLBW, very low birth weight.

The groups showed significantly different cortical thickness in areas in all cortical lobes at timepoint 2, as shown in Figure 3 and Table 3. Group differences in cortical thickness were much less widespread than those for surface area (Table 3). Thicker occipital cortex and medial frontal cortex was found bilaterally in the VLBW group, while thinning was seen in posterior temporal lobe, particularly on the left side. Left and right hemispheres showed generally the same extent of group differences, with some variation in location. Cohen’s *d* values (Figure 3D, range: −0.91 > *d* < 1.36 on left; −0.81 > *d* < 1.57 on right) indicated areas of large effect sizes in group differences. Cortical thickness results from timepoint 1 in this study using the longitudinal processing stream are presented in Supplementary Figure 2.

**Figure 4:**
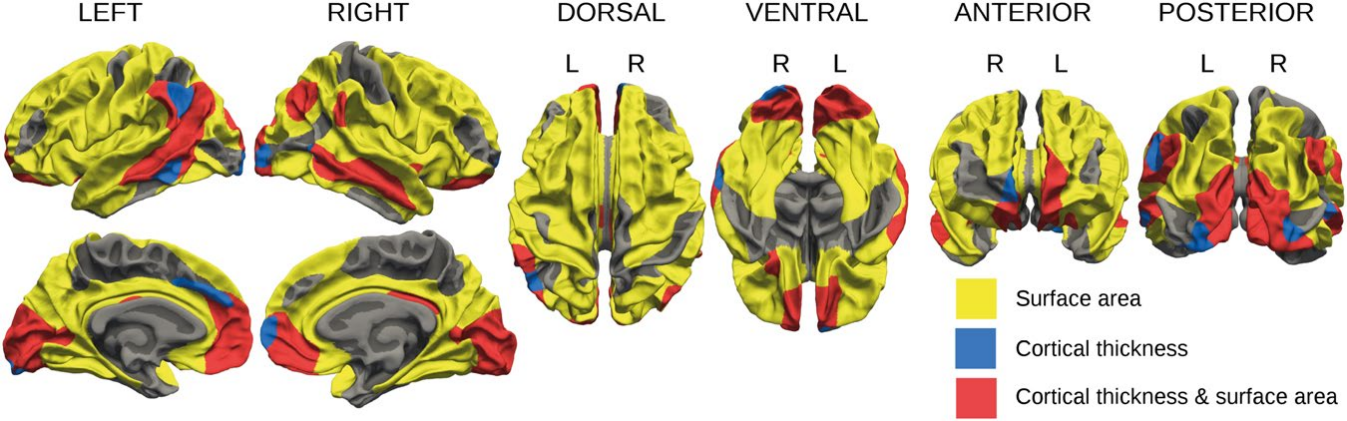
Cortical regions demonstrating significant group differences in cortical thickness (blue), surface area (yellow), and both (red) at timepoint 2.

Figure 4 summarizes cortical regions demonstrating significant group differences in cortical thickness, surface area, and both. The majority of areas that differed significantly in cortical thickness also differed in surface area (overlap shown in red). The cortical areas showing group differences in both thickness and surface area were similar bilaterally: superior temporal sulcus, angular gyrus, supramarginal gyrus, anterior cingulate, orbitofrontal cortex, cuneus, and calcarine sulcus; right hemisphere also showed overlap in the posterior cingulate, and left hemisphere showed more overlap in the superior temporal sulcus. Group differences in cortical surface area and thickness when additionally covarying for retinopathy of prematurity (Supplementary Figure 3) showed similar patterns of areas affected to those in Figures 2 and 3.

### Cortical morphometry and cognitive performance

**Figure 5:**
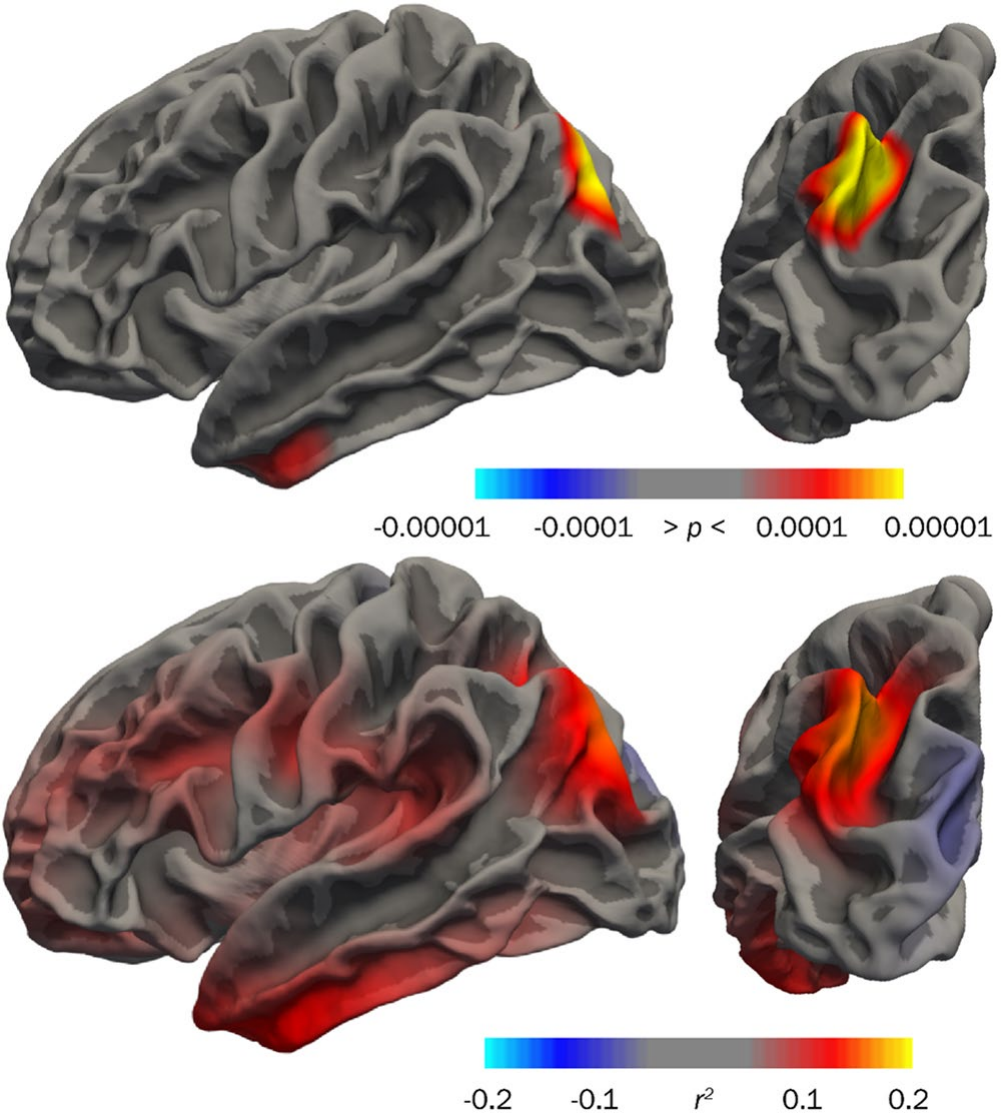
Effect size map (*r*^2^, bottom row) and *p-*map (top row) showing group × IQ interaction at timepoint 2, indicating parieto-occipital and inferior temporal regions where greater surface area was associated with higher IQ scores in the VLBW group. Left, left hemisphere lateral view; right, left hemisphere posterior view.

Two left hemisphere regions showed a group × IQ interaction in surface area at timepoint 2 (Figure 5). Significant group × IQ interactions were seen at the border of the left parietal and occipital lobes (superior occipital and transverse occipital sulci, middle occipital gyrus, angular gyrus, intraparietal sulcus in the left hemisphere) and to a lesser extent in the left inferior temporal cortex (middle temporal gyrus towards the frontal pole). Greater surface area in these regions was associated with higher IQ scores in the VLBW group (*r*^2^ ≤ 0.16). These areas also showed group × IQ interaction at timepoint 1, as previously reported^35^.

No significant group × score interactions for the executive functions were found for surface area or cortical thickness in the longitudinal or cross-sectional analyses after FDR correction.

In the control group only, longitudinal surface area changes showed a structure-function relationship with change in NEPSY statue scores, with higher scores associated with increased surface area in right superior precentral sulcus, left planum temporale, transverse temporal sulcus, posterior segment of the lateral sulcus, and cingulate gyrus posterior-ventral. For spatial span, higher scores were associated with reduced surface area in right subparietal sulcus and the marginal part of the cingulate sulcus. See Supplementary Figure 3.

## Discussion

No group × time interactions were seen in brain growth between approximately 8 and 9.3 years of age, while significant group differences in subcortical volumes and, cortical surface area, and to a lesser extent cortical thickness, persisted from timepoint 1 to 2. However, within the VLBW group, higher IQ was associated with greater surface area in left hemisphere regions of parieto-occipital and inferior temporal cortex, and perinatal health markers were to a limited extent associated with reduced volumes in right hippocampus and left thalamus. We found no group × time longitudinal interactions in morphometry for the executive function scores. The longitudinal results suggest a similar trajectory of cortical and subcortical development between preterm and term-born peers in this middle childhood window, in conjunction with evidence of altered cognitive networks in preterm-born children. This result among preterm children born in the 2000s extends our findings from a cohort born in the late 1980s, which demonstrated that VLBW preterm-born individuals and term-born peers did not show divergent developmental trajectories for cortical thickness, surface area^26^, or subcortical volumes^23^ from 15 to 20 years of age.

### Executive function and IQ interaction with morphometric development

Lower IQ was associated with reduced surface area in two cortical regions within the VLBW group (Figure 5). In the area in parieto-occipital cortex, the preterm group showed both significantly reduced surface area, as well as an added effect of IQ on surface area in the preterm group compared to term-born (i.e., increased surface area and higher IQ). This finding points to a specific region within a lateral parieto-temporal module reported to show stronger association between IQ and gray matter volume in very preterm-born adolescents/adults^22^. This area constitutes part of the proposed dorsal stream^66,67^, which is believed to be involved in visuomotor control of actions^68^. The dorsal stream is reported to be impacted in the preterm population^69^, among other clinical groups, and may be related to impairments in attention and executive function^70^, a potential explanation for its relationship to IQ scores in this study. This interesting finding may indicate specific cortical regions where surface area development reflects compensatory mechanisms used for general cognitive abilities in place by early school age.

This study did not find group × executive function score interactions for morphometry measures in early school age. These findings were somewhat unexpected, as altered morphometry has been frequently associated with differences in executive function, visual-motor skills, and cognition in long-term follow-up of preterm birth survivors^71–76^. It is possible that the relatively high functioning of the preterm-born sample in this study limited the ability to identify different structure-score associations between groups, making it difficult to comment on reorganization or alternate cortical development among preterm survivors with more reduced executive function.

Executive function encompasses encompass working memory, cognitive control, and inhibitory control/reward processing^77,78^. Prematurity can lead to a cascade of downstream impairments on cognitive performance, beginning with slower processing speed, poorer executive functioning and working memory, and finally lower achievement in math and reading^79^. Consistent with the hypothesis of Rose, *et al*.^79^, preterm-born children in this sample were indeed more likely than term-born peers to receive special services in school.

It is possible that differential trajectories linking executive function and cortical development occur earlier in life, prior to the age window assessed in this study. Rathbone *et al*.^80^ found a positive correlation between perinatal growth rate of cortical surface area and NEPSY summary score at age 6 and speculated that genetic and environmental influences during infancy are related to the development in childhood of executive function, attention, and planning capacities. Term-born and preterm-born children did not show significantly different trajectories in this study between the ages of 8 and 9.3, similar to findings of Edgin, *et al*.^10^ in children between the ages of 2 and 4. Very preterm-born children in the Edgin, *et al*.^10^ study had similar executive function performance as term-born peers, while those with white matter abnormalities showed persistent cognitive inflexibility and poor inhibitory control, underscoring the value of neuroimaging for identifying individuals at highest risk for cognitive control difficulties already by age two.

Functional neuroimaging studies have shown evidence for differences in verbal, learning, and memory cortical circuits in the preterm population^81–90^. Further multimodal analysis in this preterm/MoBa cohort combining white matter imaging with morphometry and functional imaging may be able identify specific risks for those with poorer white matter development.

### Persistent differences in cortical thickness and surface area

Both cortical thickness and surface area showed widespread cross-sectional group differences in this study as expected, with significant overlap (Figure 4), which held when taking retinopathy of prematurity into account (Supplementary Figure 2). Frontal, occipital, and temporo-parietal regions implicated in this study have previously shown the greatest cortical thickness deviation among the most immature preterm survivors (birth weight ≤ 1250 g or gestational age ≤ 28 weeks) in adolescence^73^.

This study’s findings of cortical and subcortical deficits may be due to epigenetic effects of immature birth on genes controlling growth, or the same effects due to fetal growth restriction caused by placental pathology. Preterm/VLBW-related insults to the brain may be first and foremost limited to the perinatal period, a highly plastic and vulnerable period for the immature brain^91–96^. Compared to fetuses of comparable age, preterm/VLBW infants who live *ex utero* for their “third trimester” show decreased brain growth – even in the absence of severe brain injury – suggesting that their course of brain development leading up to term age is altered^97^. Disruption in this window of plasticity may initiate or require reorganization of neural connections^29,91^.

The ages assessed in this study encompass periods of peak cortical thickness and surface area^98–100^, which are followed by pruning of experience-expectant synapses and plasticity through adolescence^101^, especially in white matter. It is likely that both neural growth and pruning mechanisms are disturbed in the preterm population, leading to the constellation of preterm brain features including both thinning and thickening of cortex and widespread surface area reduction.

The structural differences in cortex among preterms in this study may be caused by a cascading mechanism from white matter damage. While the most severe lesions including cystic periventricular leukomalacia have declined following advances in neonatal care, periventricular white matter injury is still common in the preterm-born population^15,102^. Vollmer *et al*.^103^ speculate that early disturbance of growth in white matter pathways, rather than reduced structural volumes, contribute to worse cognitive function in the preterm-born population^104,105^.

### Reduced subcortical structure volumes

The VLBW group showed persistently smaller volumes of corpus callosum, right globus pallidus, and right thalamus. Growth rates for subcortical structures (Table 2) did not differ between groups whether or not ICV was used as a covariate. Corpus callosum, hippocampus, and thalamus are particularly vulnerable in this group, and alterations in their development in the VLBW population have been linked to cognitive deficits or psychiatric symptoms^23,106–108^. Corpus callosum volume in this clinical population has been linked to IQ^17^ and executive functions^11,109,110^.

Moreover, volume associations with cognitive and perinatal markers in the preterm group were predominant in structures critical for learning, memory, and cognition. Volumes of right hippocampus and left thalamus were related to perinatal health measures in the VLBW group. Smaller corpus callosum subsegmentation volumes, especially posteriorly, showed strong correlations to receiving help at school. Posterior corpus callosum is often affected in long-term follow-up of preterm survivors, and perinatal brain injury to its connectivity may affect visual and perceptual skills^111^. Formation of deep gray matter structures, in particular the thalamus, occurs at the same time as preterm birth and is linked to development of white matter and cortex^112^. Thalamocortical fibers and projections from sensory and associative thalamus can be affected by both focal and diffuse lesions in prematurity, related to vulnerability of subplate neurons in the second and third trimesters^113,114^. These functional outcomes likely share common mechanisms with structural alterations and/or reflect cascading cognitive effects.

It is difficult to discern whether or to what extent these structural changes are compensating for impaired function in the preterm brain, or are altered for physiological reasons as part of the so-called encephalopathy of prematurity^20,115^. Moreover, reduced brain volumes may not be caused by prematurity itself, but rather postnatal events and risk factors, such as the need for prolonged supplementary oxygen, which may exert an environmental influence on brain growth^116^.

### Clinical and Classroom Implications

These results suggest that improvements in neonatal medical care and other early childhood services have buffered the neurodevelopmental impact of preterm birth with VLBW and that children born in the 2000s show more similar structure-function relationships to their term-born peers than in previous decades^36^. For example, intubation for extremely preterm neonates has been increasingly replaced with less-invasive methods of ventilation, such as increased surfactant use and early continuous positive airway pressure^102,117^.

The preterm behavioral phenotype has been described as anxious and inattentive, rather than hyperactive or disruptive, which may also mean that their cognitive difficulties may not be as readily visible in a classroom setting^20,118,119^. Promisingly, working memory training interventions have shown learning gains in preterm-born preschoolers and adolescents^120,121^. Identifying sensitive windows for “catch up” in brain function is critical for survivors of preterm birth with VLBW, given their well-documented cognitive challenges.

### Strengths and Limitations

Structural MRI can detect variation in early brain development and serve as a reference point for functional differences^122,123^. Longitudinal imaging is the only way to accurately measure structural growth and maturation and determine links between cognitive development and brain growth^124^. A strength of this study is the use of a robust longitudinal image registration protocol^55^ and a statistical analysis approach that allows for explicit modeling and analysis of within- and across-subject sources of variability in temporal covariance^64^. Following the same individuals longitudinally is a challenge for researchers, and this study includes MRI data from 2 timepoints from 120 children, with no significant difference in socioeconomic status between groups. This study included a subset of participants in the Norwegian MoBa Study, which in total recruited 108,000 births from across Norway in an extensive prospective study of health and development^38^. Future study designs could do more to incentivize continued participation to ensure a robust and representative sample.

Although group × time analyses did not show significant interactions for any of the brain structures, it is still possible that the growth rates in the two groups do differ, but either at a different age, or at a rate that was not detectable in this study. A challenge with the longitudinal cognitive assessment portion was the possibility of practice effects when administering the same test twice^125^. Moreover, the NEPSY Statue subtest is typically administered to children three to six years old but was used here with older children as part of the longitudinal design and to increase standardization across the entire age span of participants. Finally, it is possible that the preterm-born children with the most compromised executive function were not included in the study due to incomplete testing and/or poor quality neuroimaging, which would skew overall performance in the preterm group upwards.

### Basis for multimodal analysis

A natural extension of this project would be to move from correlational analysis to developing a predictive model that integrates multimodal MRI features (such as cortical thickness, surface area, subcortical volumes, white matter properties, and activation patterns) and with neuropsychological and cognitive follow-up results. Differences in the development of brain structures can parallel differences in cognitive skills and behavior^126,127^. Predictive modeling using functional and morphometric data has shown potential in estimating cognitive skills later in childhood^128^ and may be useful for the VLBW community^129^. As more neurodevelopmental predictive models using MRI are created and tested, they may prove useful for identifying children with greater need for follow-up services and education interventions^130^ or to predict potential variance in treatment outcomes^131,132^. As preterm birth and VLBW remain global health challenges, increasing our knowledge linking subtle changes in brain structures with measurable deficits in cognitive performance is an important step forward.

A future step would be to study gray and white matter changes longitudinally in the same cohort to better link white matter damage with cortical development and maturation, and evaluate how this relationship evolves starting in the neonatal period, since morphometric and diffusion parameters have been shown to follow different developmental paths^111,133–135^. While this study does not provide evidence for catch-up growth in brain structures among preterm-born individuals, it is possible that there is a critical window open earlier in childhood during which targeted interventions can stimulate cognitive development, especially in the most at-risk babies born preterm with VLBW.

## Electronic supplementary material

Supplementary material

## Data Availability

Inquiries about and requests for access to data generated and analyzed during this study should be directed to the corresponding author.
