# Supplementary material for: Trajectories of brain development in school-age children born preterm with very low birth weight
Source: Sci Rep. 2018 Oct 22;8:15553. doi: 10.1038/s41598-018-33530-8 (PMC6197262; doi:10.1038/s41598-018-33530-8)
Supplement: Supplementary file 1 — Supplementary material [file 41598_2018_33530_MOESM1_ESM.docx]

**Supplementary Material**

**Trajectories of brain development in school-age children born preterm with very low birth weight**

K. Sripada^1^*, K. J. Bjuland^2^, A. E. Sølsnes^1^, A. K. Håberg^3,4^, K. H. Grunewaldt^1,5^, G. C. Løhaugen^2^, L. M. Rimol^4,6^, J. Skranes^1,2^

^1^ Department of Clinical and Molecular Medicine, Norwegian University of Science & Technology, Trondheim, Norway;

^2^ Department of Pediatrics, Sørlandet Hospital, Arendal, Norway;

^3^ Department of Neuromedicine & Movement Science, Norwegian University of Science and Technology, Trondheim, Norway;

^4^ Department of Medical Imaging, St. Olav’s Hospital, Trondheim, Norway;

^5^ Department of Pediatrics, St. Olav’s Hospital, Trondheim, Norway;

^6^ Department of Circulation and Medical Imaging, Norwegian University of Science and Technology, Trondheim, Norway.

**Corresponding author*

**Supplemental Table 1.** Subcortical gray matter structures and corpus callosum volumes (mm³) and ICV (cm³) in VLBW and control subjects, with group differences at the two timepoints and longitudinally.

| Structure | | Timepoint 1 | | | | | | | | Timepoint 2 | | | | | | | | Longitudinal | |
| --- | --- | --- | --- | --- | --- | --- | --- | --- | --- | --- | --- | --- | --- | --- | --- | --- | --- | --- | --- |
|  |  | VLBW (n=37) | | | Control (n=116) | | | *d* | *p* | VLBW (n=30) | | | Control (n=90) | | | *d* | *p* |  |  |
|  |  | Mean | SD | 95% CI | Mean | SD | 95% CI |  |  | Mean | SD | 95% CI | Mean | SD | 95% CI |  |  | *F* | *p* |
| Corpus callosum | Total | 2363 | 365 | [2244, 2481] | 2759 | 353 | [2695, 2824] | 1.10 | <0.001* | 2462 | 386 | [2323, 2602] | 2832 | 371 | [2754, 2909] | 0.98 | <0.001* | 1.03 | 0.31 |
|  | Posterior | 644 | 121 | [605, 683] | 772 | 117 | [751, 793] | 1.08 | <0.001* | 686 | 128 | [640, 733] | 802 | 123 | [776, 828] | 0.92 | <0.001* | 0.00 | 0.97 |
|  | Mid-posterior | 285 | 70 | [262, 307] | 381 | 68 | [369, 394] | 1.40 | <0.001* | 308 | 76 | [281, 336] | 394 | 73 | [379, 409] | 1.14 | <0.001* | 0.37 | 0.54 |
|  | Central | 315 | 70 | [292, 338] | 386 | 68 | [374, 399] | 1.03 | <0.001* | 328 | 77 | [300, 356] | 401 | 74 | [385, 416] | 0.96 | <0.001* | 0.65 | 0.42 |
|  | Mid-anterior | 354 | 80 | [328, 380] | 410 | 78 | [396, 425] | 0.71 | <0.001* | 363 | 82 | [333, 392] | 417 | 79 | [401, 434] | 0.68 | 0.002 | 0.71 | 0.40 |
|  | Anterior | 765 | 121 | [725, 804] | 810 | 117 | [788, 831] | 0.38 | 0.056 | 777 | 127 | [731, 823] | 818 | 122 | [793, 844] | 0.33 | 0.131 | 0.01 | 0.92 |
| Amygdala | Left | 1308 | 139 | [1263, 1353] | 1400 | 135 | [1375, 1425] | 0.67 | 0.001 | 1370 | 139 | [1320, 1420] | 1445 | 134 | [1417, 1473] | 0.55 | 0.013 | 0.18 | 0.68 |
|  | Right | 1406 | 147 | [1358, 1454] | 1443 | 142 | [1417, 1469] | 0.26 | 0.187 | 1455 | 162 | [1397, 1514] | 1477 | 156 | [1445, 1510] | 0.14 | 0.532 | 0.08 | 0.77 |
| Caudate | Left | 4023 | 462 | [3873, 4174] | 4084 | 447 | [4001, 4166] | 0.13 | 0.498 | 4120 | 504 | [3937, 4302] | 4135 | 486 | [4034, 4237] | 0.03 | 0.886 | 0.19 | 0.67 |
|  | Right | 4252 | 563 | [4069, 4435] | 4255 | 545 | [4155, 4355] | 0.01 | 0.978 | 4293 | 583 | [4082, 4503] | 4284 | 561 | [4167, 4401] | -0.01 | 0.946 | 0.09 | 0.77 |
| Cerebellar cortex | Left | 55456 | 4982 | [53838, 57075] | 55839 | 4822 | [54954, 56724] | 0.08 | 0.689 | 56604 | 4965 | [54809, 58400] | 55784 | 4780 | [54786, 56782] | -0.17 | 0.443 | 1.52 | 0.22 |
|  | Right | 56916 | 5082 | [55265, 58567] | 56476 | 4918 | [55574, 57378] | -0.09 | 0.652 | 57824 | 5094 | [55981, 59666] | 56445 | 4905 | [55421, 57469] | -0.28 | 0.210 | 1.83 | 0.18 |
| Globus pallidus | Left | 1980 | 260 | [1896, 2065] | 2098 | 252 | [2052, 2145] | 0.46 | 0.019 | 2019 | 273 | [1920, 2118] | 2141 | 262 | [2086, 2196] | 0.46 | 0.039 | 0.01 | 0.93 |
|  | Right | 1739 | 198 | [1674, 1803] | 1927 | 191 | [1892, 1962] | 0.97 | <0.001* | 1829 | 205 | [1755, 1903] | 1980 | 197 | [1939, 2021] | 0.75 | 0.001* | 1.44 | 0.23 |
| Hippocampus | Left | 3634 | 376 | [3512, 3756] | 3907 | 364 | [3841, 3974] | 0.74 | <0.001* | 3702 | 406 | [3555, 3849] | 3968 | 390 | [3887, 4050] | 0.67 | 0.003 | 1.03 | 0.31 |
|  | Right | 3785 | 326 | [3679, 3891] | 4026 | 316 | [3968, 4084] | 0.75 | <0.001* | 3894 | 344 | [3770, 4018] | 4078 | 331 | [4009, 4147] | 0.54 | 0.014 | 1.32 | 0.25 |
| Nucleus accumbens | Left | 668 | 118 | [629, 706] | 688 | 114 | [667, 708] | 0.17 | 0.379 | 673 | 115 | [632, 715] | 708 | 111 | [685, 731] | 0.31 | 0.165 | 1.72 | 0.19 |
|  | Right | 656 | 96 | [625, 687] | 702 | 93 | [685, 719] | 0.49 | 0.014 | 691 | 103 | [653, 728] | 718 | 100 | [697, 738] | 0.27 | 0.228 | 0.05 | 0.83 |
| Putamen | Left | 6109 | 582 | [5920, 6299] | 6315 | 563 | [6212, 6418] | 0.36 | 0.067 | 6234 | 580 | [6024, 6444] | 6430 | 558 | [6314, 6547] | 0.34 | 0.118 | 2.35 | 0.13 |
|  | Right | 6174 | 569 | [5989, 6358] | 6372 | 550 | [6271, 6473] | 0.35 | 0.071 | 6245 | 579 | [6036, 6454] | 6458 | 557 | [6342, 6574] | 0.37 | 0.089 | 0.50 | 0.48 |
| Thalamus | Left | 7397 | 500 | [7234, 7560] | 7787 | 484 | [7698, 7876] | 0.79 | <0.001* | 7622 | 516 | [7436, 7809] | 7962 | 496 | [7859, 8066] | 0.67 | 0.003 | 0.73 | 0.39 |
|  | Right | 7127 | 509 | [6961, 7292] | 7633 | 493 | [7542, 7723] | 1.01 | <0.001* | 7297 | 544 | [7100, 7493] | 7748 | 524 | [7638, 7857] | 0.84 | <0.001* | 0.80 | 0.37 |
| Ventricular system | | 25831 | 15339 | [20848, 30814] | 12066 | 14845 | [9342, 14790] | -0.91 | <0.001* | 21581 | 8220 | 8221 | 12668 | 7914 | 7914 | -1.10 | <0.001* | 0.22 | 0.64 |
| ICV (cm³) | | 1416 | 118 | [1378, 1454] | 1502 | 117 | [1481, 1524] | 1.03 | <0.001* | 1432 | 120 | 121 | 1526 | 119 | 119 | 1.11 | <0.001* | 5.77 | 0.02 |

Group differences tested using the general linear model, controlled for ICV, age at scan, sex; ICV only corrected for age at scan and sex. Holm–Bonferroni step-down used to determine significance threshold based on 24 comparisons; significant results denoted by *. *Abbreviations*: CI: confidence interval; ICV: intracranial volume; VLBW: very low birth weight.

**
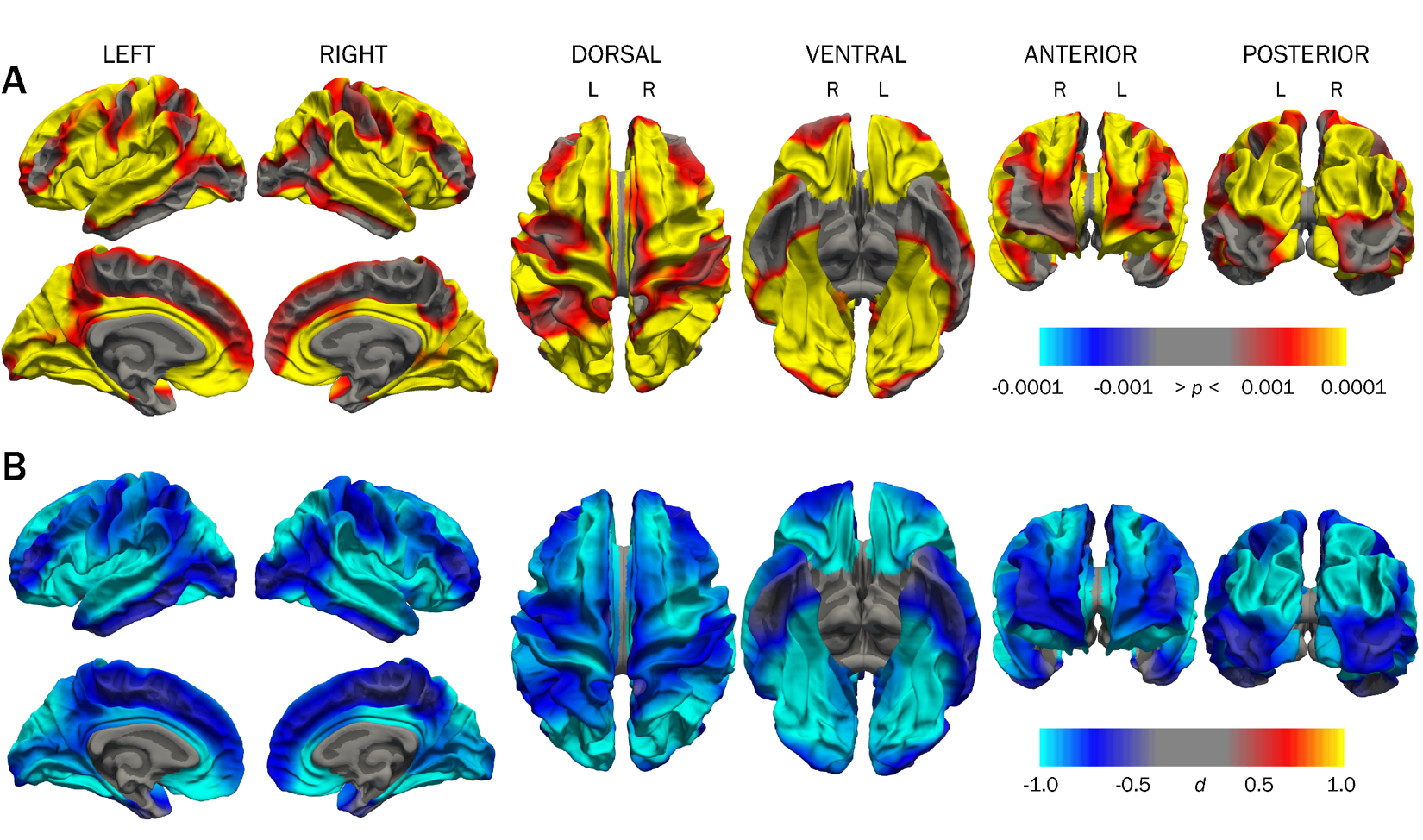
**

**Supplementary Figure 1**. Cortical surface area group differences between VLBW and control groups at timepoint 1. Row A shows *p-*maps and row B shows effect size. The *p-*maps were produced from GLM models fitted at each location (vertex) across the cortical surface, with cortical area as the dependent variable and group as the independent variable, co-varying for sex and age at scan. The *p*-maps were thresholded to yield an expected 5% FDR across both hemispheres. In the effect size maps, blue represents areas of reduced surface area in the VLBW. *Abbreviations*: *d*: Cohen’s *d*; FDR: false discovery rate; VLBW, very low birth weight.


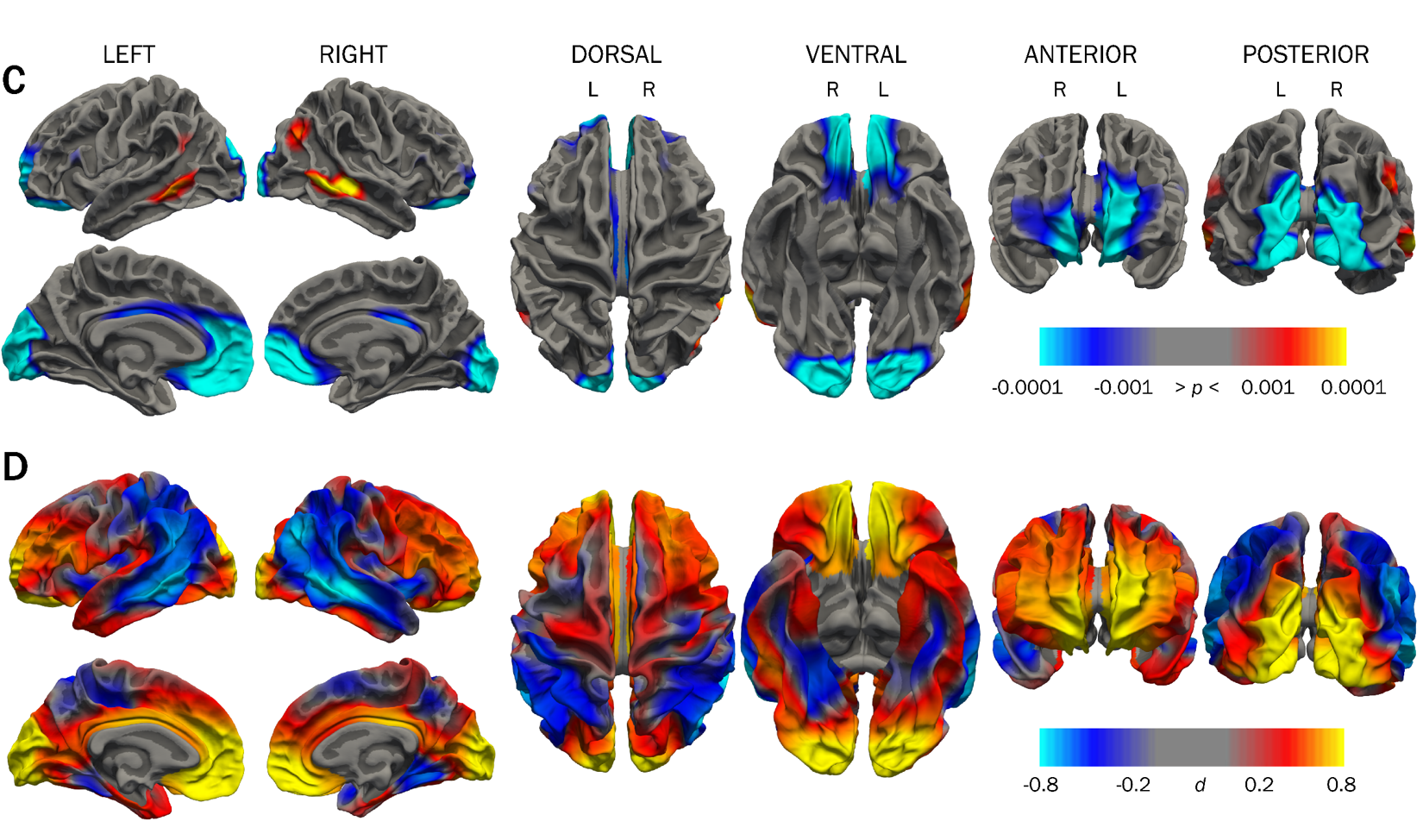


**Supplementary Figure 2**. Cortical thickness group differences between VLBW and control groups at timepoint 1. Row C shows *p-*maps and row D shows effect size. The *p-*maps were produced from GLM models fitted at each vertex across the cortical surface, with cortical thickness as the dependent variable and group as the independent variable, co-varying for sex and age at scan. The *p*-maps were thresholded to yield an expected 5% FDR across both hemispheres. In the effect size maps, red-yellow color represents areas of increased thickness in the VLBW group, while blue represents areas of cortical thinning in the VLBW group. *Abbreviations*: *d*: Cohen’s *d*; FDR: false discovery rate; VLBW, very low birth weight.


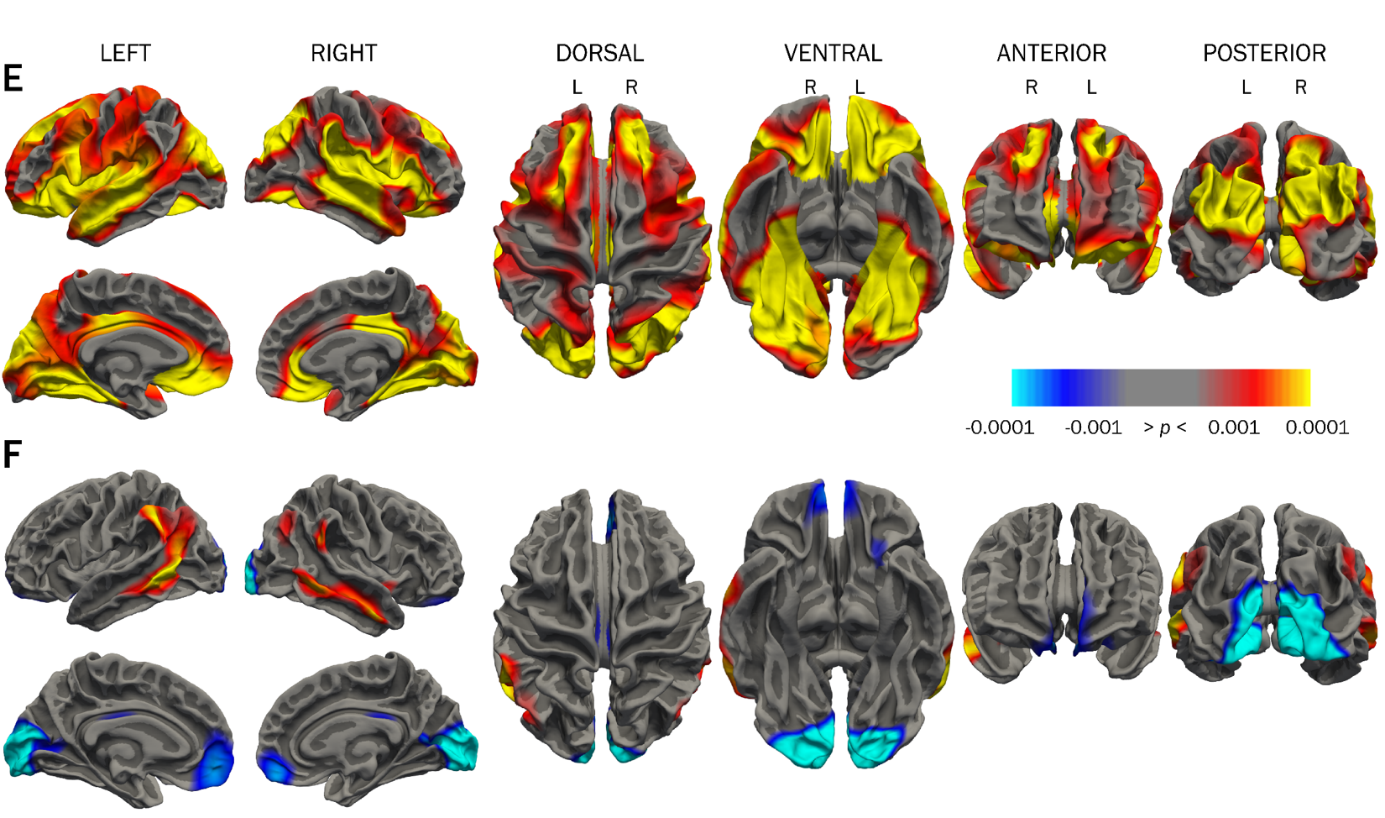


**Supplementary Figure 3.** Group differences between VLBW and control groups including retinopathy of prematurity as a covariate at timepoint 2. Row E shows *p-*maps of group difference in cortical surface area and row F shows the same for cortical thickness. The *p-*maps were produced from GLM models fitted at each vertex across the cortical surface, with cortical measure (area or thickness) as the dependent variable and group as the independent variable, co-varying for sex, age at scan, and retinopathy of prematurity. The *p*-maps were thresholded to yield an expected 5% FDR across both hemispheres. *Abbreviations*: FDR: false discovery rate; VLBW, very low birth weight.


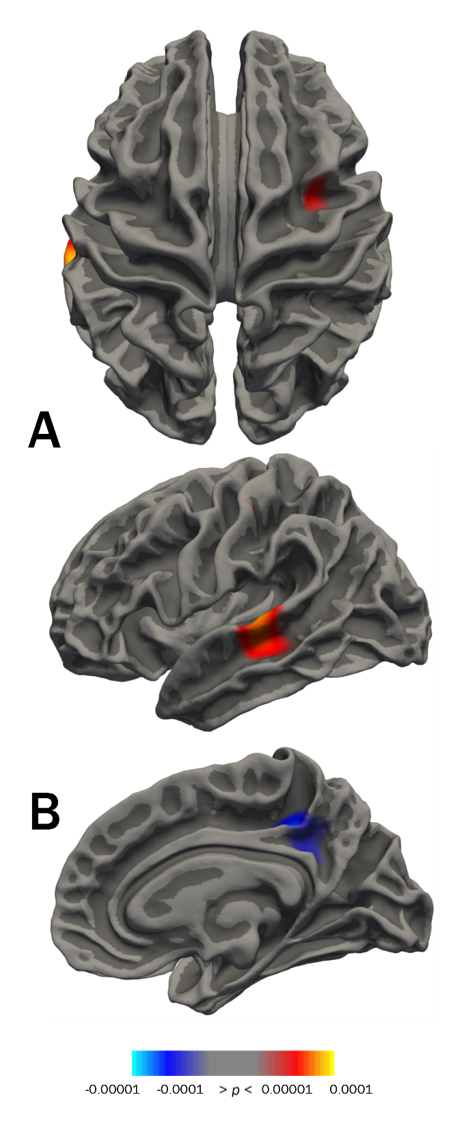


**Supplementary Figure 3.** Longitudinal *p*–maps of structure-function relationships in the term-born group between cortical surface area and change in statue score (A – top two images) and spatial span (B) based on linear mixed effects models. A indicates increased surface area is associated with higher statue scores in the cortical areas in yellow-red. B indicates increased surface area is associated with lower spatial span scores in the cortical area in blue.
